# Targeting Aurora A Kinase (AAK) in Platinum-Resistant High Grade Serous Ovarian Cancer

**DOI:** 10.3389/fonc.2020.01354

**Published:** 2020-08-19

**Authors:** Ram N. Ganapathi, Eric J. Norris, Ashley P. Sutker, Kaitlin E. Klotz, Mahrukh K. Ganapathi

**Affiliations:** Carolinas Medical Center, Levine Cancer Institute, Charlotte, NC, United States

**Keywords:** aurora A kinase, high grade serous ovarian cancer, platinum-resistance, alisertib, *BRCA*

## Abstract

Aurora A kinase (AAK) involved in G_2_-M transition is functionally involved in centrosome maturation and maintaining an active spindle assembly checkpoint. We tested the hypothesis that in platinum-taxane resistant high grade serous ovarian cancer (HGSOC) inhibition of AAK involved in G_2_-M transition would enhance the anti-tumor activity of cisplatin (CP) or paclitaxel (PT). Using HGSOC cell lines from platinum-taxane refractory patients that do not harbor *BRCA1/2* mutations, we tested the anti-tumor activity of CP, or PT alone or in combination with the AAK inhibitor alisertib (AL). Treatment with CP for 3 h or PT for 6 h followed sequentially by AL for 48 h led to a significant decrease in cell survival (*p* < 0.001) compared to treatment with either drug alone in HGSOC cells but not in immortalized normal human ovarian surface epithelium or normal human fallopian tube secretory epithelium cells. The treatment with CP or PT followed by AL also led to a significant increase in reactive oxygen species (*p* < 0.05), apoptosis (*p* < 0.001) and accumulation of cells in G_2_/M that was accompanied by a modest increase in expression of AAK. Downregulation of AAK, but not aurora B kinase, with targeted siRNAs also significantly enhanced apoptosis by CP or PT, suggesting that AL specifically targeted AAK. In summary, in HGSOC without *BRCA1/2* mutations, CP, or PT resistance can potentially be circumvented by sequential treatment with AL that inhibits AAK involved in G_2_-M transition.

## Introduction

Epithelial ovarian cancer (EOC) is the most lethal gynecologic cancer and comprises variousdistinct subtypes that differ in genetic drivers, histologic features, and clinical outcome (1). High-grade serous ovarian carcinoma (HGSOC) is the most common and aggressive subtype of EOC and current standard of care for patients with HGSOC includes platinum-based adjuvant or neo-adjuvant chemotherapy (1–3). Despite initial response rates of >70% following surgery and first-line chemotherapy, tumor recurrence develops in ~70–80% of these patients (2, 3). Additionally, response rates to second-line therapy decreases with each recurrence due to the development of drug resistance (2, 3).

Although several studies have investigated the molecular basis of chemoresistance to platinum/taxane chemotherapy in HGSOC, few effective treatment strategies have been identified or validated for use in clinical practice. For patients with *BRCA1/2* mutations or homologous recombination deficiency (HRD), personalized second-line treatment strategies with inhibitors of poly (adenosine diphosphate ribose) polymerase (PARP) has resulted in remarkable enhancement of disease-free survival (4). However, in patients with recurrent HGSOC without *BRCA* mutations or no HRD, few effective therapeutic options are currently available (4). Since, platinum and taxane effects on DNA damage or microtubule polymerization, respectively, lead to accumulation/arrest of tumor cells in the G_2_/M phase of the cell cycle and subsequent cell death (5, 6), a potential mechanism for drug resistance could involve escape of tumor cells from G_2_/M arrest and cell death. Thus, in the absence of *BRCA1*/*BRCA2* mutations or HRD in recurrent HGSOC, strategies that manipulate the DNA damage checkpoint or G_2_-M transition could help alleviate drug resistance.

Aurora A kinase (AAK) regulates G_2_-M transition by promoting centrosome maturation and mitotic spindle assembly (7). Overexpression of AAK is observed in many aneuploid tumors and is an important predictor of patient prognosis (8, 9). Because of the oncogenic potential of AAK, which has been shown to involve interaction with other oncogenic proteins, selective inhibitors of AAK have been developed (8, 9). Alisertib (AL, MLN8237) is a potent and selective inhibitor of AAK (10, 11), which has shown promising anti-tumor activity in several pre-clinical tumor models and in clinical trials either as a single agent or in combination with other active anti-cancer drugs (11). Pre-clinical studies have demonstrated the potential for inhibiting AAK to improve efficacy of cisplatin or taxane chemotherapy (12–14). Results from a recent randomized clinical trial report encouraging progression-free survival with the combination of paclitaxel and AL treatment compared to paclitaxel alone in recurrent ovarian cancer (15).

Based on few therapeutic options for recurrent platinum-taxane resistant HGSOC without *BRCA1*/*BRCA2* mutations or HRD, we sought to test the hypothesis that inhibiting aurora A kinase (AAK) with AL and interfering with G_2_-M transition would enhance the anti-tumor activity of cisplatin (CP) or paclitaxel (PT). Using cell culture models of recurrent HGSOC without *BRCA* mutations that were established from patients clinically refractory to platinum/taxane therapy, we demonstrate that the efficacy of AL treatment with CP or PT which is sequence dependent, results in enhanced growth inhibition, generation of reactive oxygen species (ROS), increased accumulation of cells in G_2_/M phase and apoptosis in tumor compared to normal cells.

## Materials and Methods

### Cell Lines

The HGSOC OC2 cell line was developed at the Cleveland Clinic (16) and UPN 251 (17) was a gift from Bristol Myers Squibb. PE04 cells were obtained from Sigma Aldrich, St. Louis, MO. The OC2, PEO4, and UPN251 cells have wild type *BRCA1/BRCA*, based on analysis with Ion AmpliSeq *BRCA1* and *BRCA2* panel (Thermo Fisher Scientific, Waltham, MA), were from patients clinically refractory to cisplatin and/or paclitaxel (16–18). The OC2, PEO4, and UPN251 cells were authenticated based on STR analysis. Immortalized human ovarian surface epithelial (HOSE) cells (19) were a gift from Dr. H. Katabuchi, Kumamoto University, Kumamoto, Japan and the FT246 human immortalized fallopian tube secretory epithelial cells (20) were a gift from Dr. Ronny Drapkin, University of Pennsylvania, Perleman School of Medicine, Philadelphia, PA. The OC2, PEO4, and UPN251 cells were cultured in RPMI1640 medium, supplemented with 10% fetal bovine serum (FBS) and 2 mM L-glutamine. HOSE cells were maintained in 1:1 DMEM/Ham's F12 Medium supplemented with 2 mM glutamine and 10% FBS. The FT cells were maintained in 1:1 DMEM/Ham's F12 supplemented with 2 mM L-glutamine and 2% Ultroser™. All cultures were maintained at 37°C in a humidified 5% CO_2_ plus 95% air atmosphere. Cell culture medium was obtained from Thermo Fisher Scientific, Waltham, MA; FBS was from Atlanta Biologicals, Norcross, GA and Ultroser™ from Pall Corporation, Port Washington, NY. Cisplatin (CP) injectable patient formulation was obtained from the pharmacy, paclitaxel (PT), and alisertib (AL) were obtained as 10 mM stock solutions in DMSO from Selleckchem, Houston, TX.

### Drug Treatment

To determine the dose of CP, PT, and AL to be used in this study, we initially carried out a dose response for each drug and used a dose that resulted in ~30–40% cell kill when used as single agents. This choice of drug concentrations was based on determination of dose response and a pharmacologically achievable drug concentration. In addition, the concentration of cisplatin or paclitaxel chosen to test the interaction with alisertib led to cell proliferation following removal of drug with no post-treatment and this was not observed with alisertib post-treatment. While higher concentrations of drug that are not clinically achievable can potentially overcome resistance, they are not useful to determine interaction with alisertib due to excessive cytotoxicity. The experimental protocol involved sequential treatment with the combination of CP or PT with AL. To test sequence dependency cells were either: (a) pre-treated with CP for 3 h or PT for 6 h followed by AL for 48 h or (b) pre-treatment with AL (48 h) followed by CP (3 h) or PT (6 h). For both sequence protocols, cells were allowed to recover in drug-free medium for an additional 96–120 h (~3–4 cell doublings for the HGSOC cells) to determine cytotoxicity, while apoptosis, cell cycle traverse perturbations and production of ROS were determined at the end of treatment.

### Determination of Cytotoxicity, Apoptosis, ROS, and Cell Cycle Traverse

Drug treatment related cytotoxicity was evaluated by determining cell counts in a TC20 automated cell counter (Bio-Rad, Hercules, CA) with trypan blue. All cell counts were carried out following recovery of drug treated cells for 120 h (~3–4 cell doublings) in drug-free medium. Apoptosis was determined by fluorescence microscopy in cells stained with Hoechst 33,342 (70 μg/ml) and propidium iodide (100 μg/ml) at 37°C for 15 min (21). The stained cells (control and treated) were viewed in a fluorescence microscope with the appropriate filters (excitation 350 nm/emission 460 nm) to visualize simultaneously the blue fluorescence from Hoechst 33,342 and the red fluorescence from propidium iodide. Normal viable cells fluorescent blue within the nucleus, and the apoptotic cells show condensation of chromatin and formation of small masses of varying sizes. Necrotic cells stain pink, but these cells are swollen, and the chromatin is not condensed and fragmented as in apoptotic cells. Apoptotic cells were scored by counting a total of 300–400 control or treated cells in each experiment to calculate the % apoptotic cells (21). To assess the effect of treatment with cisplatin and/or alisertib on total intracellular ROS levels, we utilized the fluorescent ROS indicator 5-(and 6-)-chloromethyl-2′, 7′-dichlorodihydrofluorescein diacetate and acetyl ester (CM-H_2_DCFDA, Thermo Fisher Scientific, Waltham, MA) as previously described (22, 23). Cells were pre-treated with CP for 3 h followed by AL for 48 h. At the end of treatment, cells were recovered by trypsinization and re-suspended in phenol red-free RPMI1640 media containing 5 μM CM-H_2_DCFDA. Following 30 min incubation at 37°C, cells were washed twice with ice cold Hank's Balanced Salt Solution (HBSS) and re-suspended in 1 mL of HBSS for FACS analysis. Cellular fluorescent intensity (excitation 488 nm, emission 520 nm) was acquired using a FACSCalibur (BD Biosciences) flow cytometer. Average fluorescent intensity was calculated using WinList software (Verity Software House, Topsham, ME). Flow cytometry for cell cycle traverse perturbations were carried out after staining with propidium iodide as described earlier (21) and cell cycle phase distribution analysis used ModFit LT 5.0 software (Verity Software House, Topsham, ME).

### Downregulation of Aurora A- and Aurora B-Kinase

Downregulation of AAK or aurora B kinase (ABK) with Silencer Select siRNA (ThermoFisher, Waltham MA) was carried out to determine selectivity of AL for inhibiting AAK or ABK. Cells in 6 well-plates were treated with CP for 3 h or PT for 6 h. Following drug treatment, culture medium was removed, replaced with drug-free medium, and transfected with 5 nM of 21-mer Silencer Select siRNA for AAK or ABK using Lipofectamine RNAiMAX transfection reagent (ThermoFisher, Waltham MA). After 72 h of exposure to the siRNA, cells were harvested for extraction of RNA and to determine apoptosis. Downregulation of AAK or ABK was determined using Taqman primers and RT-PCR.

### Western Blotting

Cells following treatment were lysed using radioimmunoprecipitation Assay (RIPA) buffer and centrifuged at 100,000 × *g* for 1 h at 4°C. The supernatant was collected, and protein concentration was determined using the Bradford Assay (Biorad, Hercules, CA, USA). Twenty micrograms of protein were separated by SDS-PAGE according to the BOLT™ western blotting protocol (ThermoFisher Scientific). Proteins were transferred to PVDF membranes and blocked with 5% non-fat dry milk in TBS/0.05% TWEEN 20 buffer for 1 h at room temperature.

Membranes were incubated at the dilution indicated for the following primary antibodies: Aurora A kinase, dilution 1–3 μg/ml (Thermo Fisher catalog# 45-8900), c-Myc, dilution 1:1,000 (Thermo Fisher, catalog# 10828-1-AP), N-Myc, dilution 1:1,000 (Thermo Fisher catalog# PA5-14287 and beta actin peroxidase labeled (Sigma catalog# A3584) overnight at 4°, washed 3×, and incubated for 1 h at room temperature with appropriate secondary antibody. Proteins were visualized using Amersham ECL Prime (GE Healthcare Life Sciences, Marlborough, MA) and imaged using UVP GelDoc-it Imaging Station and software (UVP, Jena, Germany).

Experiments were carried out in triplicate unless otherwise stated as described in figure legends. Data from experiments evaluating cytotoxicity, apoptosis, and ROS was statistically analyzed by two-way analysis of variance using SigmaPlot 12.5 software.

## Results

### Enhancement of Cisplatin and Paclitaxel Cytotoxicity by Alisertib in HGSOC Cell Lines is Dependent on Treatment Sequence

**Figure 1:**
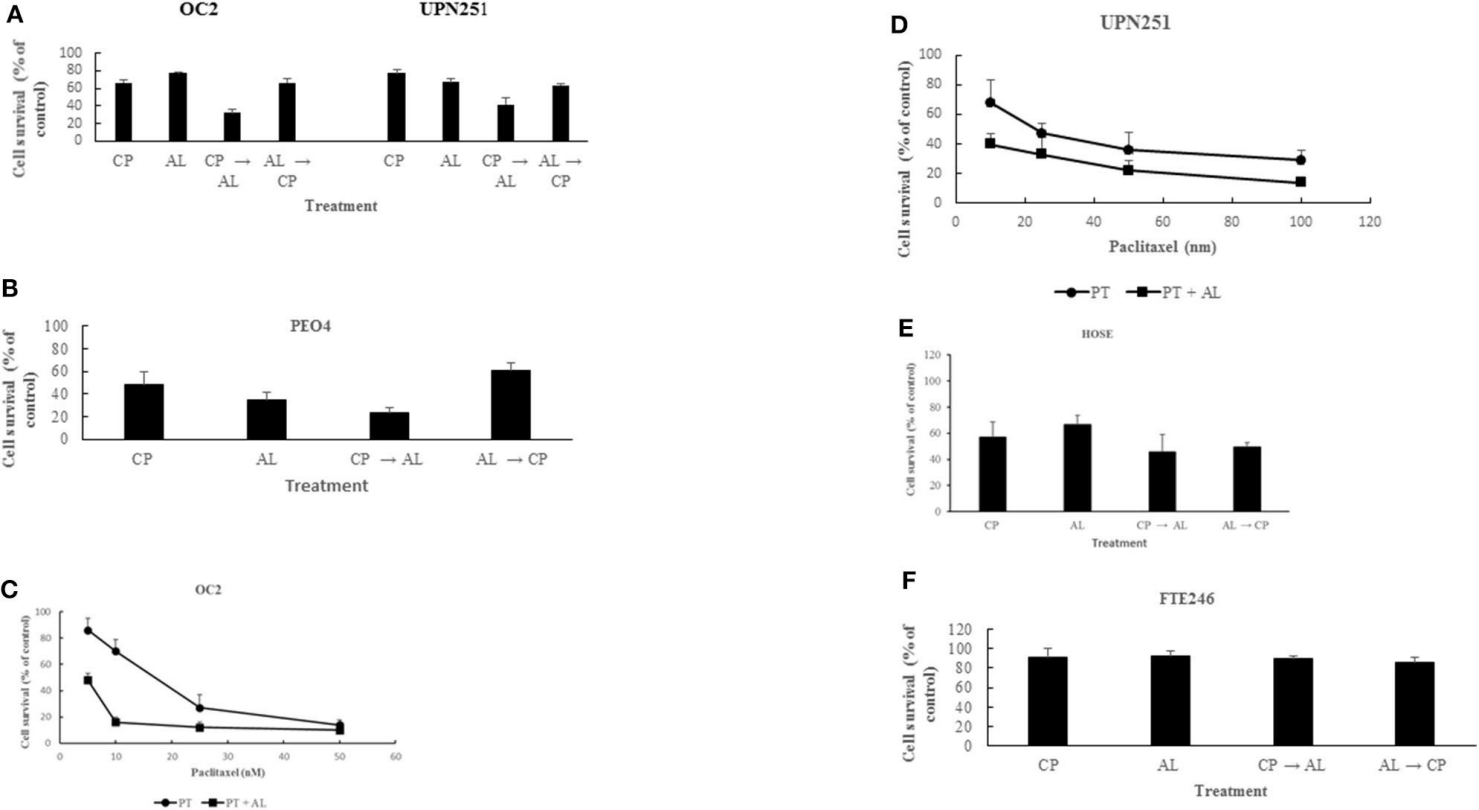
Synergistic enhancement of cisplatin and paclitaxel cytotoxicity by alisertib. **(A)** OC2 and UPN251 cells were treated with 2.5 or 1 μM cisplatin (CP), respectively, for 3 h and 50 nM alisertib for 48 h either before or after treatment with CP. Following drug treatment cells were re-incubated in drug-free medium for 120 h before determination of cell counts. CP → AL was significantly different from AL → CP, CP, or AL treatment (*p* < 0.001). **(B)** PEO4 cells were treated with 2.5 μM CP and 50 nM AL. Treatment and evaluation parameters like **(A)**. CP → AL was significantly different from AL → CP, CP, or AL treatment (*p* < 0.002). **(C,D)** OC2 and UPN251 cells were treated with indicated concentrations of paclitaxel (PT) alone for 6 h or PT for 6 h followed by alisertib (AL) for 48 h. Following drug treatment cells were re-incubated in drug-free medium for 120 h before determination of cell counts. Data are mean ± standard deviation from at least triplicate experiments. Treatment with PT followed by AL significantly different from treatment with PT alone (*p* < 0.001 at 5–25 nM PT in OC2 and *p* < 0.05 at 10–100 nM PT in UPN251). **(E)** HOSE and **(F)** FT246 cells were treated with 2.5 μM (CP) for 3 h and 50 nM alisertib for 48 h either before or after treatment with CP. Following drug treatment cells were re-incubated in drug-free medium for 120 h before determination of cell counts. CP → AL, AL → CP, CP, or AL treatment were not significantly different (*p* > 0.05). Data are mean ± standard deviation from at least triplicate experiments.

Treatment with CP for 3 h followed by AL for 48 h led to synergistic cell kill (*p* < 0.001) in 3 different HGSCOC cell lines (Figures 1A,B). In contrast, pre-treatment for 48 h with AL followed by cisplatin for 3 h led to anti-proliferative effects that was not different from treatment with CP or AL alone in all 3 HGSOC cell lines. These results suggest that potentiation of CP induced cytotoxicity by AL is sequence dependent. Similar to the results with CP, treatment with PT for 6 h followed by AL for 48 h also led to a synergistic enhancement of PT induced cytotoxicity over a wide range of drug concentrations (*p* < 0.001 at 5–25 nM PT in OC2 and *p* < 0.05 at 10–100 nM PT in UPN251) in 2 different HGSOC cell lines (Figures 1C,D). However, in cells pre-treated with AL followed by PT, cytotoxic effects were either additive or antagonistic (data not shown). Unlike results with the HGSOC cells, in both normal HOSE and FT246 cells neither synergism nor antagonism was observed with CP treatment either prior to or after AL exposure (Figures 1E,F). The rationale for using the HOSE and FT246 cells was to determine whether the synergistic cytotoxicity with alisertib in tumor cells was also observed with normal cells. This data is useful since toxicity to normal tissues is a problem with synergistic effects of combination therapy. Further, toxicity to other normal tissues can be more comprehensively evaluated using *in vivo* pre-clinical models. Reduced cytotoxicity in the FT246 compared to HOSE cells could be related to silencing of p53 in the FT246 cells.

### Alisertib Enhances ROS Accumulation and Apoptosis With Cisplatin Treatment

**Figure 2:**
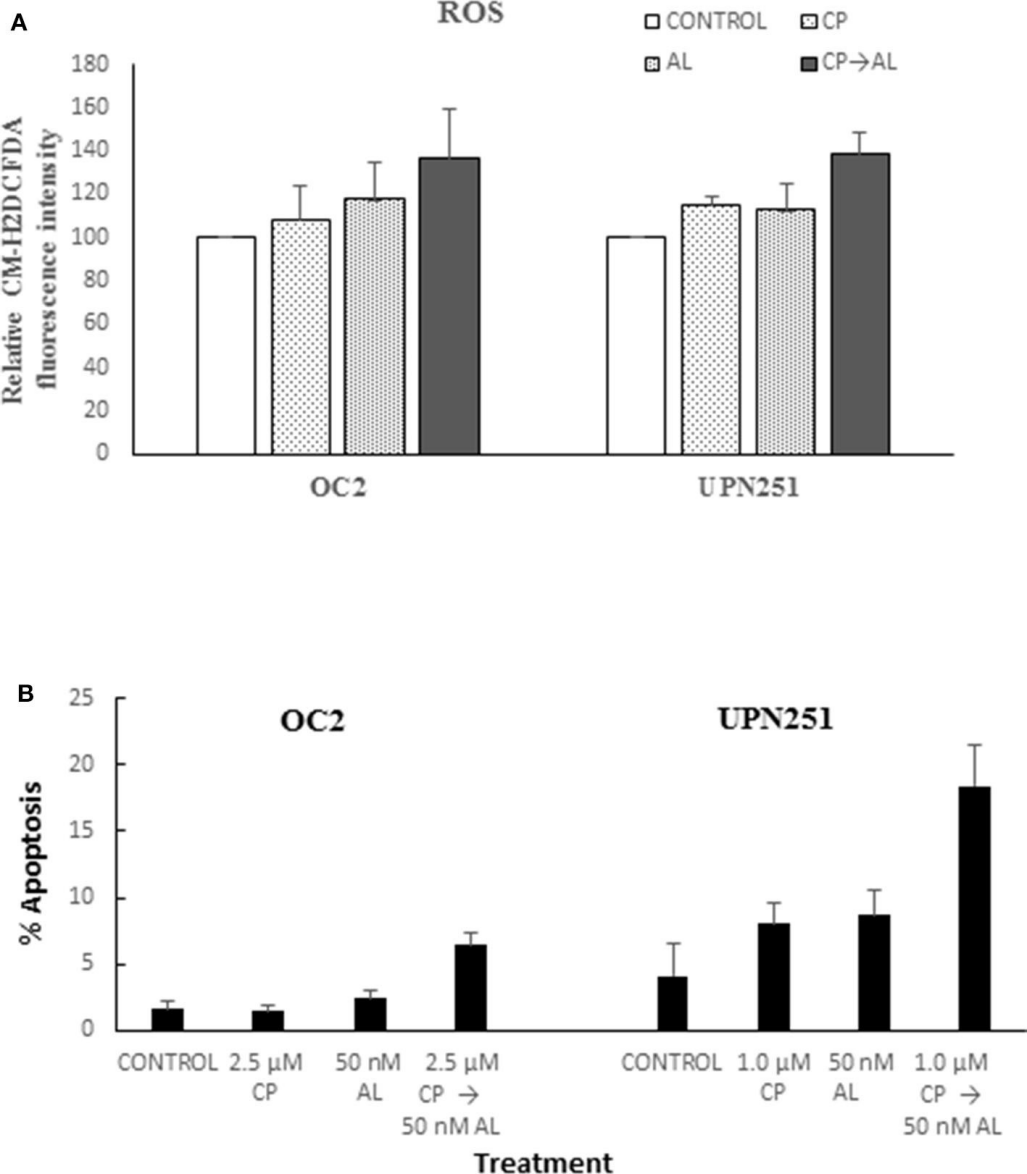
Alisertib enhances ROS accumulation and apoptosis with cisplatin treatment. **(A)** OC2 and UPN251 cells were treated with 2.5 or 1 μM cisplatin (CP), respectively, for 3 h followed by 50 nM alisertib for 48 h. Following treatment cells were stained with the fluorescent ROS indicator 5-(and 6-)-chloromethyl-2′, 7′-dichlorodihydrofluorescein diacetate and acetyl ester (CM-H_2_DCFDA, Thermo Fisher Scientific, Waltham, MA) as previously described (23). Cellular fluorescent intensity (excitation 488 nm, emission 520 nm) was acquired using a FACSCalibur (BD Biosciences) flow cytometer. Average fluorescent intensity was calculated using WinList software (Verity Software House, Topsham, ME). Treatment with CP followed by AL led to increased ROS production in both OC2 (*p* < 0.05) and UP251 (*p* < 0.01) cells compared to the untreated control and treatment with either CP or AL alone. **(B)** OC2 and UPN251 cells were treated with cisplatin (CP), for 3 h followed by 50 nM alisertib for 48 h. Following treatment cells were stained with Hoechst 33,342 (70 μg/ml) and propidium iodide (100 μg/ml) at 37°C for 15 min and apoptotic cells quantified by fluorescence microscopy (21). A minimum of 300–400 cells were counted in each experiment. Treatment with CP followed by AL led to significantly increased apoptosis in both OC2 and UP251 (*p* < 0.001) cells compared to the untreated control and treatment either CP or AL alone. Data for ROS and apoptosis are mean ± standard deviation from at least triplicate experiments.

To determine mechanisms involved in the potentiation of CP cytotoxicity by AL we evaluated generation of ROS based on CM-H_2_DCFDA fluorescence, since the cytotoxic effects of cisplatin are associated with rapid accumulation of ROS (24, 25). As outlined in Figure 2A treatment with CP followed by AL led to increased ROS production in both OC2 (*p* < 0.05) and UP251 (*p* < 0.01) cells as compared to the untreated control, CP or AL treated cells. Similar increase in ROS was not observed with CP treatment followed by AL in normal FT246 or HOSE cells (data not shown). Further, this increased generation of ROS with CP treatment followed by AL was accompanied by a 2.5–3-fold increase in apoptosis (*p* < 0.001) compared to control, AL, or CP alone in OC2 and UPN251 cells (Figure 2B).

### Accumulation of Cells in G_2_/M Is Increased by Alisertib With Cisplatin Treatment

**Figure 3:**
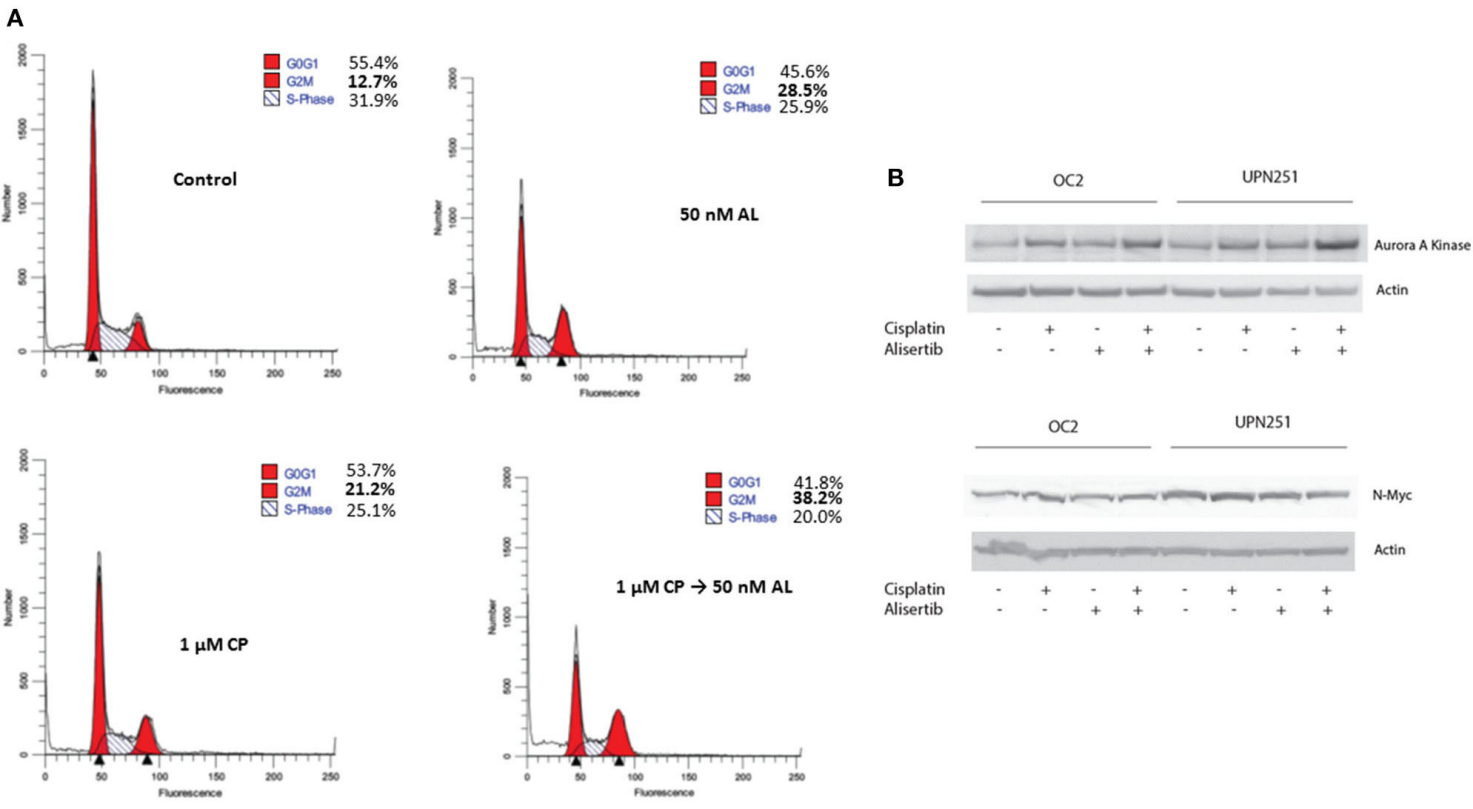
Alisertib enhances accumulation of cells in G_2_/M following cisplatin treatment. **(A)** UPN251 cells were treated with cisplatin (CP), for 3 h followed by 50 nM alisertib for 48 h. Following treatment cells were stained with and propidium iodide and analyzed by flow cytometry. **(B)** OC2 and UPN251 cells were treated as in Figure 2A and cell lysates prepared following drug treatment. Lysates were analyzed by SDS-PAGE and probed for expression of AAK, N-myc, and actin.

Since CP treatment followed by AL led to enhanced cytotoxicity, we evaluated cell cycle traverse perturbations of control and treated cells by flow cytometry. As outlined in Figure 3A, representative data with UPN251 cells treated with CP followed by AL led to enhanced accumulation of cells in the G_2_/M phase, compared to the control or treatment with CP or AL alone. In lysates of cells treated with CP, AL, or CP + AL and analyzed by SDS-PAGE, a modest increase in AAK protein levels was observed in OC2 or UPN251 cells treated with CP followed by AL compared to the untreated control, CP or AL treatment alone (Figure 3B). The increase in AAK protein levels with CP + AL treatment agreest with the increased accumulation of cells in the G_2_/M phase of the cell cycle. No reproducible increase or decrease in N-myc (Figure 3B) or c-myc protein levels was observed (data not shown) in OC2 or UPN251 cells for the various treatment conditions. Overall, treatment with CP followed by AL led to increased ROS and apoptosis as well as an increase in AAK protein levels and accumulation of cells in G_2_/M phase of the cell cycle.

### Alisertib Targets Aurora A Kinase for Potentiation of Cisplatin and Paclitaxel Apoptosis

**Figure 4:**
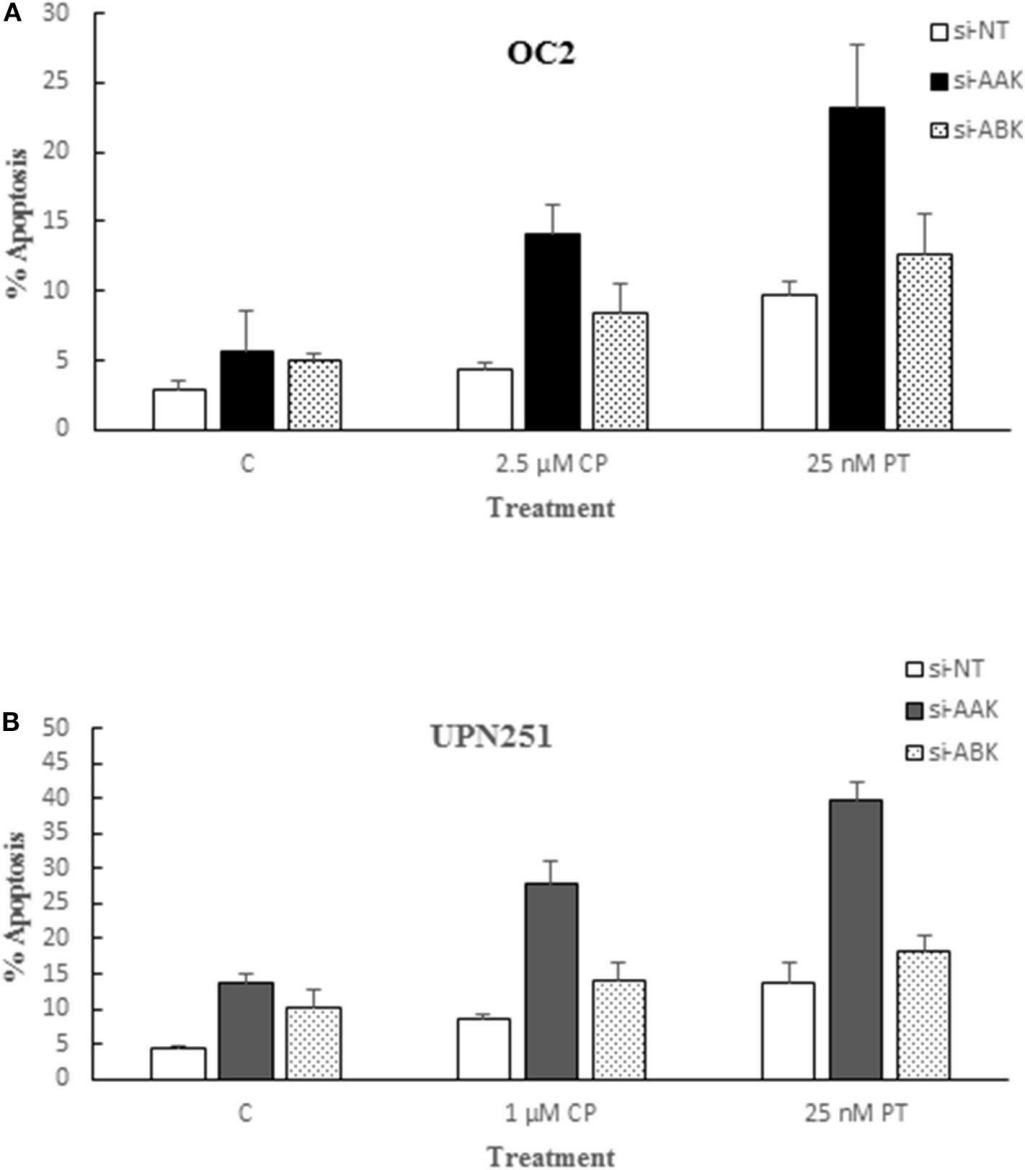
Alisertib targets aurora A kinase for potentiation of cisplatin and paclitaxel induced apoptosis. OC2 **(A)** and UPN2561 **(B)** cells were treated with indicated concentrations of cisplatin for 3 h or paclitaxel for 6 h followed by removal of drug and transfection with a non-targeting siRNA (NT) or siRNA targeting AAK or ABK for 72 h. Apoptosis at the end of siRNA exposure was determined as described earlier for Figure 2B. Data are mean ± standard deviation from at least triplicate experiments. siAAK compared to NT (OC2–CP 2.5-fold; PT 4.1-fold; UPN251- CP 2.0-fold; PT 2.9-fold) vs. siABK compared to NT (OC2–CP 1.7-fold; PT 2.5-fold; UPN251- CP 1.4-fold; PT 1.8-fold).

Since the aurora kinase family is comprised of three known members and AL, albeit specific for AAK, could also target other family members, we examined the effect of down regulating AAK and Aurora B Kinase (ABK) with specific siRNAs in OC2 and UPN251 cells on CP and PT induced apoptosis. In replicate experiments, downregulation of AAK in OC2 and UP251 was 81 ± 7 and 68 ± 12%, respectively. The results revealed that while down regulation of AAK and ABK both enhanced apoptosis in CP and PT treated OC2 and UPN251 HGSOC cells, the degree of potentiation was significantly higher when AAK was downregulated (Figures 4A,B; OC2—CP 2.5-fold; PT 4.1-fold; UPN251- CP 2.0-fold; PT 2.9-fold). In contrast, following downregulation of ABK (Figures 4A,B) CP- and PT-induced apoptosis was enhanced to a lesser extent than that observed following down regulation with AAK (OC2—CP 1.7-fold; PT 2.5-fold; UPN251- CP 1.4-fold; PT 1.8-fold) using targeted siRNA. Analysis of interaction between siRNA targeting AAK for OC2 (*p* < 0.001), UPN251 (*p* = 0.004) for CP treatment and for OC2 (*p* < 0.015), UPN251 (*p* < 0.001) with PT treatment suggested synergism. In contrast similar analysis between siRNA targeting ABK OC2 (*p* = 0.201), UPN251 (*p* = 0.943) for CP treatment and OC2 (*p* = 0.956), UPN251 (*p* = 0.698) for PT treatment, suggest no synergism. These results affirm the role of AAK, since the enhanced cytotoxicity with 50 nM AL and CP or PT treatment (Figure 1) was observed at a clinically achievable concentration reported to be selective for inhibition of AAK, (26).

## Discussion

A response rate >70% for primary HGSOC with adjuvant platinum and taxane chemotherapy following cytoreductive surgery is overshadowed by equally high recurrence rates, which continues to be a major clinical problem. Maintenance therapy for improving progression-free survival with PARP inhibitors holds great promise for personalized therapy in women with HRD (4). However, few options are available for patients with no-HRD and failure of 2nd line treatment can lead to reduced progression-free survival with subsequent therapy. Thus, in this study we opted to explore the G_2_/M checkpoint that is essential for invoking cytotoxic effects of CP or PT. Indeed, in HGSOC cell lines without *BRCA1/BRCA2* mutations from patients clinically refractory to platinum/taxane treatment we demonstrate that following CP or PT treatment the sequential use of the AAK inhibitor AL at clinically achievable drug concentrations (26) leads to synergistic cell kill.

Aurora kinases are key regulators of mitosis, which are overexpressed in several tumors leading to genomic instability. (27–29). Therefore, aurora kinases are important therapeutic targets and several inhibitors targeting AAK and ABK have been developed. AL, an inhibitor of AAK, has been evaluated for anti-tumor activity as a single agent and in combination therapy in pre-clinical and clinical trials (10–12, 26). While single agent activity has been disappointing, results of a recent randomized clinical trial examining combination of PT and AL are encouraging for progression-free survival (15). Further, it has been suggested that AL has the potential for reversing CP resistance in tumors overexpressing AAK (14). Indeed, the present studies carried out in HGSOC derived from patients that were clinically refractory to cisplatin and/or paclitaxel support the beneficial effects of the sequential combination of CP or PT with AL, only when CP or PT are used prior to treatment with AL. This is noteworthy since these cell lines, which do not have *BRCA1/BRCA2* mutation, are generally not responsive to the alternative PARP inhibition therapies. Although combining PARP inhibitors with AL could potentially be efficacious based on the suggestion that inhibition of AAK mimics “BRCAness” (30), our preliminary results (data not shown) examining the combination of AL and olaparib in PEO4 HGSOC cells with wild type *BRCA* (18) did not lead to potentiation of cytotoxicity. At best, an additive effect was observed. This finding suggests that in BRCA WT tumors the combination of AL with CP or PT may be more effective that the combination of AL with PARP inhibitors.

Since AAK is an important component of the DNA damage response/repair pathway that may be activated following DNA damage induced by CP and PT, especially in resistant cells, inhibition of AAK catalytic activity would be expected to blunt repair and mitotic progression and activate cell death. Indeed, it has been reported that following treatment with the DNA cross linking agent mitomycin C, phosphorylation of FANCA at S165 by AAK activates the Fanconi anemia/BRCA repair pathway (31). However, downregulation of AAK or expression of mutant S165A FANCA sensitizes tumor cells to mitomycin C. Since CP is a DNA cross linking agent and the OC2 as well as UPN251 cells do not harbor *BRCA1/BRCA2* mutations, the enhanced cytotoxicity with CP/AL could be related to inhibition of AAK activation of the Fanconi anemia/BRCA repair pathway by AL. The enhanced cytotoxicity with PT followed by AL is possibly linked to inhibitory effects of AL on G_2_M transition, since it has been shown that the anti-proliferative effects of PT related to microtubule polymerization and arrest at metaphase-anaphase can be overcome by AAK leading to resistance (29, 32). While combination therapy with AL has not considered cell cycle phase specificity or G_2_/M checkpoint failure, results in this study demonstrate that the efficacy of AL to potentiate cytotoxicity is sequence dependent, suggesting that G_2_/M block induced by AL pre-treatment could inhibit subsequent cellular drug effects of CP or PT that contribute to a cytotoxic response. Whereas, post-treatment with AL preserves the G_2_/M block induced by CP or PT and leads to increased expression of AAK, the repair pathway is likely impaired because of inhibition of AAK by AL. Thus, in the absence of DNA repair, arrest of cells in G_2_/M would likely result in mitotic catastrophe/augmented apoptotic response. The apoptotic response is likely associated with generation of ROS. Our finding demonstrating that downregulation of AAK is superior at enhancing the cytotoxicity of CP and PT than downregulation of ABK is of interest, since clinical development of ABK inhibitors has been abandoned due to lack of efficacy (33). The sequential use of CP or PT followed by AL offers a useful clinical treatment paradigm to enhance anti-tumor activity and reduce systemic toxicity compared to simultaneous combination therapy.

In summary, we provide compelling new data demonstrating that sequential treatment with CP or PT exposure followed by AL for targeting AAK may be a clinically active treatment option for recurrent platinum-sensitive or platinum-resistant HGSOC without *BRCA1*/*BRCA2* mutations. Unlike HRD positive tumors that are intrinsically sensitive to CP or PARP inhibitors, mechanistic basis for regulation of G_2_/M checkpoint control with or without HRD in recurrent platinum-sensitive or platinum-resistant HGSOC should be explored to develop novel therapeutic strategies.

## Data Availability Statement

The raw data supporting the conclusions of this article will be made available by the authors, without undue reservation.

## Author Contributions

RG and MG conceptualized the project and wrote the manuscript. RG, EN, AS, KK, and MG contributed to the design and methodology for the experiments performed and were involved in review and editing the manuscript. All authors contributed to the article and approved the submitted version.

## Conflict of Interest

The authors declare that the research was conducted in the absence of any commercial or financial relationships that could be construed as a potential conflict of interest.
